# The concept of procedural law regarding the implementation of collective agreements with legal certainty in termination of employment in Indonesia

**DOI:** 10.1016/j.heliyon.2021.e06690

**Published:** 2021-04-05

**Authors:** Salahuddin Gaffar, Agus Mulya Karsona, Yani Pujiwati, Indra Perwira

**Affiliations:** Faculty of Law, Universitas Padjadjaran, Bandung, West Java, Indonesia

**Keywords:** Bipartite, Legal certainty, Collective agreement, Termination of employment

## Abstract

Bipartite negotiations are an effort to resolve disputes that the parties must take in advance, as mandated in Article 3 of Law Number 2 of 2004. Suppose there is an agreement in bipartite negotiations. In that case, these agreements are written into a collective contract that is then registered with an industrial relations court to obtain a registration certificate. However, collective agreements that have been agreed upon and registered and have executorial legal force are still being sued by one of the court parties. The purpose of this study is to obtain an overview and analyze the concept of procedural law regarding the implementation of collective agreements with legal certainty in terminating employment in Indonesia. This research is legal research using a statutory approach, a conceptual approach, and a case approach—the collection of material through the literature study method, with primary and secondary legal materials. Furthermore, the traditional fabric is studied and analyzed by the approaches used in this study to answer legal issues in this study. In this article, the author offers 2 (two) procedural law concepts regarding the implementation of collective agreements. First, a lawsuit for disputes that have been resolved through a cooperative agreement may not be accepted by the court, and the judge does not need to process the case further. Still, a file research process is sufficient to explore the main problem of the parties, if the problem is related to one of the parties breaking the promise by referring to the recorded evidence issued by the same court, the Panel of Judges is sufficient to hold a deliberation and only determine with a single judge that the case has been resolved and choose the order for execution. The second concept is that the lawsuit is still being processed. Still, it is only continued until the interim decision stage if, at the initial examination stage, it is known that the dispute has been resolved through a collective agreement. This is far more effective, efficient and fair, and provides legal certainty for the parties.

## 1 Introduction

Humans, as living creatures show two aspects that cannot be separated from one another. The first aspect is humans as individual beings and other humans' factors as members of society in togetherness with other individual humans (Charda, 2017). One of the goals of humans living in a community is to meet the various needs of their life, including clothing, food and shelter. To meet these needs, humans are required to work. In doing human work, one human being has a relationship (Merita and Haryadi, 2020). This relationship is called industrial relations. Industrial relation is a system of relationships in the form of actors producing goods or services consisting of elements of entrepreneurs, workers and government based on the values of Pancasila and the 1945 Constitution (Kunarti, 2009).

In human relations, disputes or disputes are always possible. Even considering that the legal subject is a human being and a legal entity, more and more parties are involved. With the increasingly complex nature of life in society, the scope of incidents or disputes is getting wider, including those often a concern in labour law, namely the issue of industrial relations disputes. Industrial relations disputes usually occur between workers/labourers and employers or between workers ' organizations/labour organizations and employers' organizations. The types of industrial relations disputes regulated in the laws and regulations, namely: disputes over rights, interests, employment termination, and conflicts between trade unions in one company. Of the various types of disputes, the most important is how the solution is to be resolved to be truly objective and fair (Husni, 2004) (see Table 1).

**Table 1:** Data on number of cases of industrial relations disputes that ended layoffs.

No	Year	Number of layoffs
1	2014	77.700
2	2015	48.800
3	2016	12.800
4	2017	9.800
5	2018	3.400
6	2019	45.000
7	2020	3.600.000 (most of the impact of the Covid-19 Pandemic)

Ministry of Manpower (2021) (Processed).

Data from the Ministry of Manpower claims that the number of layoffs in Indonesia has continued to decline since 2014. In 2018 the number of releases had been signed up to 3.4 thousand workers or decreased by 95.67 per cent. However, this figure rose again in 2019 to 45 thousand releases. This figure also jumped up to 3.6 million layoffs. This is the impact of the Covid-19 pandemic that has occurred since the beginning of 2020.

The parties themselves can resolve the settlement of industrial relations disputes in Indonesia. If the parties cannot fix it, it will only be determined by the presence of a third party, either provided by the state or the parties themselves. Laws and regulations governing the settlement of industrial relations dispute through a long road led to the birth of Law Number 2 of 2004 concerning the Settlement of Industrial Relations Disputes. In this law, dispute resolution between workers and employers can be pursued through court (litigation) and outside court (non-litigation). Through court (litigation), the settlement mechanism is taken to the industrial relations court and the Supreme Court. Meanwhile, the settlement mechanism outside the court (non-litigation) can be pursued using bipartite negotiation, mediation, conciliation and arbitration.

Settlement of industrial relations disputes in this law is obliged to make efforts to settle disputes by way of deliberation to reach a consensus as stipulated in Article 3 of Law Number 2 of 2004 called a bipartite settlement effort. If there is an agreement between the two parties in the process, then the contract is outlined in a collective agreement that is binding for both parties and registered with the court. However, efforts to settle disputes through bipartite included in the cooperative agreement need to be reconsidered on its effectiveness and its relationship to legal certainty for the parties. The collective agreement that has been agreed upon and registered and has executive legal force is still being sued by one of the court parties.

## 2 Method

The research method used is a standard research method that aims to find solutions to legal issues. According to Marzuki (2014), Legal Research is a know-how activity in legal action, not just know-about. As a know-how activity, legal research is carried out to solve legal issues at hand. The approach to the problem used is the statutory approach (statute approach), conceptual approach (conceptual approach), and the case approach (case approach). The statutory process is carried out by examining all laws and regulations relating to the legal issue being handled. This approach is taken to find the ratio legis and ontological basis for the birth of the law. Meanwhile, the conceptual approach departs from the views and doctrines developed in the science of law. By studying the theories and philosophies in the science of law, researchers will find ideas that give birth to legal notions, legal concepts, and legal principles relevant to the issues at hand.

Legal materials used in the research are primary and secondary standard materials. Primary traditional materials referred to in the 1945 Constitution, Civil Code, Republic of Indonesia Law Number 40 of 2007 concerning Limited Liability Companies, Law of the Republic of Indonesia Number 13 of 2003 concerning Manpower, Law of the Republic of Indonesia Number 2 of 2004 concerning Settlement of Industrial Relations Disputes, and other laws and regulations related to the legal issues discussed. Secondary traditional materials include materials that support primary conventional materials such as textbooks, articles in various magazines, scientific journals in the field of law, and other supporting sources.

## 3 Result and discussion

### 3.1 The concept of procedural law in termination of employment in Indonesia

National development, especially in the human resources sector, is directed to the greatest possible extent for the active community's prosperity and welfare. Therefore, labour law must guarantee legal certainty, the value of justice, principles of benefit, order, protection and law enforcement. Along with the development in the human resources sector, many business actors are improving themselves after the economic and monetary crisis to wake up from a nightmare, as well as being exposed to the waves of the global financial crisis that hit Southeast Asia, in which Indonesia cannot be separated from these waves. The government to overcome the global economic situation together with the community, especially business actors, is one of the main reasons for stabilizing the economy and maintaining monetary balance and avoiding the bankruptcy of most companies which affects most of the fate of workers which leads to termination of employment (Arliman, 2018).

Termination of employment is one type of industrial relations dispute that often arises in the job. This can be seen from the data submitted by the Indonesian Trade Union Association (ASPEK), which states that from the beginning of 2019 to August 2019 there have been terminations of 3,000 (three thousand) workers (Kontan, 2020). Furthermore, in 2020, based on the results of a survey by Saiful Mujani Research and Consulting (SMRC), it was stated that around 29 million workers experienced layoffs (Liputan6.com, 2020).

The company cites several reasons for terminating employment, ranging from financial difficulties, efficiency, and employee performance dissatisfaction. However, the workforce is always a weak party when faced with employers who are potent parties. As a party that is still considered vulnerable, it is not uncommon for workers to ever experience injustice when dealing with company interests. Given this, the law must protect workers due to the imbalance of power between employers and workers. The law is expected to provide an equal position to both parties to create balance and justice in deciding employment termination for workers (Prameswari and Handayani, 2020).

In essence, a dispute over employment termination occurs because of a disagreement with what the parties promised in the implementation of an agreement between workers and employers. This cannot be tolerated because it will interfere with the rights and obligations and the interests of the parties that have been regulated in statutory regulations (Ferricha, 2019). Mariam Darus Badrulzaman stated that to lead to avoid disputes or disputes, it is necessary to have legal provisions or rules that must be obeyed by community members. Among other things, the law functions as a community servant, a guard so that society's movement runs smoothly, and its interests can be fulfilled. Dispute resolution is a mechanism adopted by the parties to resolve disputes over differences in the parties' interests (Sufiarina and Fakhirah, 2014).

Settlement of employment termination disputes has initially been regulated in Law Number 22 the Year 1957 concerning Labor Dispute Resolution, more regulated explicitly in Law Number 12 the Year 1964 concerning Termination of Employment in Private Companies. The process is quite long, starting with P4D (Regional Labor Dispute Settlement Committee), then being compared to P4P (Central Labor Dispute Settlement Committee). Both processes are under the auspices of the Ministry of Manpower (Tobing, 2018). If the parties cannot accept the P4P decision, then they can make efforts to cancel the decision through the State Administrative Court (PTUN) and arrive at the cancellation of the decision to the Supreme Court of the Republic of Indonesia (Khakim, 2009).

The aforementioned statutory regulations have not resulted in a fast, precise, fair and low-cost settlement of disputes. The above principle cannot accommodate developments because individual workers/labourers' rights have not been adjusted. In this law, industrial relations dispute settlement only regulates disputes over ownership and interests collectively, while the settlement of conflicts individually has not been accommodated (Charda, 2017). Therefore, Law Number 2 of 2004 concerning the Settlement of Industrial Relations Disputes was issued. In this law, if an industrial relations dispute occurs, it can be resolved in 2 (two) ways, namely through the litigation (court) or through non-litigation (outside the court). The litigation path starts from the district court's industrial relations court to the supreme court level (Zulaichah, 2019).

The non-litigation settlement is the first attempt in settling industrial relations disputes, while litigation settlement is the last resort if the first attempt does not materialize. This is a mandate in the labour laws and regulations that the payment of industrial relations disputes must be carried out by employers and workers/labourers or workers/labour unions by deliberation to reach a consensus (Fuqoha, 2020). Akbar Pradima (2013) also expressed the same thing, who stated that industrial relations disputes outside the court are a mandatory settlement stage. Dispute resolution through non-litigation channels can be resolved by, among others: bipartite negotiation, mediation, conciliation and arbitration.

Of the 4 (four) non-litigation ways of resolving disputes, settlement using bipartite negotiation are the primary step that must be carried out in resolving labour disputes by employers, workers and labour unions (especially problems with the termination of employment) as a deliberative step to resolve industrial relations disputes. For consensus (Ridwan and Nurhakim, 2020). This is following the mandate of Article 3 paragraph (1) of Law Number 2 of 2004, which reads "industrial relations disputes must be sought for resolution through deliberative bipartite negotiations to reach consensus."

Negotiation is an effort made through discussion, persuasive action/persuasion, and compromise to reach an agreement with other parties regarding certain/several specific problems and a process in which parties have the same or conflicting interests.) meet and talk intending to reach a mutually agreed agreement (Manurung, 2018). Bipartite negotiations are negotiations conducted between employers or a combination of employers and workers or workers/labour unions or other workers' unions in a disputing company. In this negotiation, both parties to the dispute attempt to resolve the dispute without a third party (Nader and Todd, 1978).

Given the definition of bipartite negotiation above, this is the same as settling civil disputes in general, known as negotiation. Based on this, bipartite negotiations are classified as alternative dispute resolution. Considering that the bipartite negotiation is a non-litigation solution, the bipartite negotiation emphasizes deliberation in solving problems. In essence, contemplation is a process of listening to each other with an attitude of accepting mutual opinions and desires based on volunteerism between the parties (Suadi, 2017).

The bipartite negotiations that occur are expected to produce a win-win solution. In the negotiation process in bipartite bargaining, to make negotiations effective and reach an agreement that benefits the parties, the conditions that affect include: (a) the parties negotiating voluntarily based on full awareness, (b) the parties are ready to deal, (c) have authority to make decisions, (d) have relatively balanced strength to create interdependence, (e) have the willingness to solve problems (Nugrogo, 2015).

Dispute settlement through bipartite must be settled no later than 30 (thirty) working days from the start date of negotiations. If within 30 (thirty) days, one of the parties refuses to conduct negotiations or has conducted negotiations but does not reach an agreement, then the bipartite negotiation is deemed a failure. When bipartite talks fail, the meal will continue with the mediation or conciliation or arbitration stages. However, if there is an agreement that makes peace for both parties, a collective agreement will be made (Suryamah, 2016). According to Wilson Bangun (2017), a cooperative agreement is a negotiation process between two parties. Workers' representatives and employers' representatives reach an agreement in the hope of getting better results without harming the parties.

The success of bipartite negotiations and the existence and strength of collective agreement law when referring to article 7 of Law Number 2 of 2004 states as follows:

a. If deliberation can reach a settlement agreement, a collective agreement is signed by the parties;
b. Collective agreement is binding and becomes law and must be implemented by the parties;
c. Collective agreement must be registered by the parties at the industrial relations court at the district court in the territory where the parties entered into a collective agreement;
d. Collective agreements that have been registered are given a certificate of proof of collective agreement registration and are an integral part of the cooperative agreement;
e. If the collective agreement is not carried out by one of the parties, then the injured party may submit a request for execution to the industrial relations court at the district court in the area of the collective agreement registered to get an order of implementation; and
f. In case the applicant for execution is domiciled outside the district court where the collective agreement is registered. The applicant for performance can submit a request for implementation through the industrial relations court at the district court in the area of domicile of the execution applicant to be forwarded to the industrial relations court at the district court which is competent to carry out the execution.

### 3.2 The concept of a joint agreement with legal certainty

The parties' collective agreement, especially in settlement of industrial relations disputes in bipartite negotiations as referred to in Article 7 paragraph (1) of Law Number 2 of 2004 as a form of peace against legal issues that have occurred. Collective agreements that exist in the form of contracts, so that the stages of formation and implementation are the same as the stages of deals in general, namely: (a) the pre-contractual stage, namely the preparatory planning stage, including the bargaining process in determining the agreement, (b) the contractual stage as the stage of the agreement formation, (c) the post-contractual stage as the stage of implementing the agreement (Kesuma and dan Vijayantera, 2020).

A joint agreement is reviewed in the principle of agreement law in the direction of freedom of contract, which gives freedom to determine agreement clauses at the pre-contractual stage and the contractual stage aimed at peace. This is because freedom of contract is an essential principle for individuals in developing themselves in their personal and social life in society. Some experts emphasize that the principle of freedom of contract is part of human rights that must be respected (Harianto, 2016).

Collective agreements are binding and become legal and must be implemented by the parties following pacta sunt servanda (Nuroini, 2015). The agreement's critical power is equivalent to the law made by the state between the legislature and the executive, and whoever breaks it can be held accountable before the law. This is known as pacta sunt servanda, where an agreement made legally is valid as law for those who make it (Hadi, 2019). This is regulated in Article 1338 of the Civil Code. However, when the pacta sunt servanda is put into practice, it requires various interpretations and adjustments. Because the pacta sunt servanda is only a basic theory (grand theory) (Fuady, 2013).

The theory of pacta sunt servanda, which Hugo Grotius teaches, is universal in that the container for binding a contract is none other than natural law itself. Mariam Darus Badrulzaman and Kompilasi (2001) states that the parties' involvement in an agreement is limited to what was agreed upon and other elements as long as custom and morally appropriate demands. Since the Greek era, when Plato and Aristotle to Rome have been made aware that the principle of propriety is indispensable to maintain the implementation of laws, demand justice (Hadi, 2019).

Collective agreements that have been made in the process of being implemented as mentioned in Article 7 paragraph (3) of Law Number 2 of 2004 are required to be registered with the industrial relations court at the local district court to obtain a certificate of proof of collective agreement registration. The obligation to write a collective agreement as regulated in these provisions does not give legal consequences to the cooperative agreement if it is not registered with the court. This means that collective agreements that are not written in court are still considered valid contracts following civil law provisions and have legal force and are binding to be implemented (Kesuma and dan Vijayantera, 2020).

The difference between a collective agreement that is registered and one that is not written lies in the traditional strength of the cooperative agreement, namely, the collective agreement that has been made and signed and registered has executive legal power so that if there is a party who violates, the injured party can request execution to court (Sejati, 2018). Requests for performance can be submitted directly to the court without waiting for a court ruling with permanent legal force (Sari et al., 2018). This is as regulated in Article 7 paragraph (5) and (6) of Law Number 2 of 2004.

However, in practice, collective agreements that have been agreed upon and registered and have executorial legal force are still being sued in court by one of the parties. Ironically, the court even accepted the lawsuit against the collective agreement and decided it differently as happened in the case of disputes over the termination of employment between Frigoglass Indonesia Company against Iin Maizar Supriatna who was questioned by the Bandung industrial relations court. The conflict has been resolved through bipartite negotiations and has obtained a collective agreement that has been registered with the industrial relations court. The result is indeed a paradox. The Panel of Judges at the Industrial Relations Court continues the trial until a decision is issued with a different verdict from the agreed collective agreement.

Apart from the above cases, another case that shows a collective agreement that is still being challenged in court is the dispute over termination of employment between SMM Company against 13 former workers. In this case, Before ending, SMM Company held a meeting with 37 workers who will be dismissed (including 13 ex-workers who are serving as extortionists) to discuss layoffs and the amount of severance pay. During the meeting, there was an agreement which was stated in the form of a collective agreement. However, 13 workers continued to file a lawsuit against the Pekanbaru Industrial Relations Court's cooperative agreement on the pretext that the severance pay was not following statutory regulations. The Pekanbaru Industrial Relations Court judge granted the 13 workers' lawsuit by ignoring the facts of the collective agreement that had been made (Nuroini, 2015).

If you look at the provisions of Article 7 paragraph (2) of Law Number 2 of 2004 in conjunction with Article 1338 of the Civil Code, as explained above, the agreement that has been made should act as law for the parties. Law must provide certainty, justice and benefits (Rato, 2010). Legal certainty can be realized through excellent and explicit norms in the form of legislation. In statutory regulations, there are no rules regarding whether or not it is permissible to file a lawsuit for damages if the collective agreement has been registered in an industrial relations court and has a cooperative agreement registration certificate. Reflecting on the above case, a new legal breakthrough is needed to provide legal certainty to the parties, because the collective agreement that has been made will be useless if it is still being sued in court.

The concept of procedural law regarding the implementation of collective agreements that the author offers in this study focuses on the process of dispute resolution through a cooperative agreement by the parties, as the main finding is that it should be in conditions where the collective agreement has executive legal force. The parties cannot challenge the contract to the industrial relations court for legal certainty and justice. The author offers 2 (two) procedural law concepts regarding the implementation of a collective agreement, namely, first, the dispute does not need to be examined through the process of suing until the final decision, but a file research process is sufficient to explore the main issues of the parties if the findings are related to one of the parties. Breaking the promise by referring to the recording evidence issued by the same court, the Panel of Judges can immediately hold a deliberation and only determine with a single judge that the case has been completed and determine the order for execution. This pattern is a kind of dismissal process at the state administrative court. Second, this pattern becomes an alternative if the court sticks to the principle of not rejecting the case (curia novit), so it doesn't matter why the lawsuit is still being processed. However, the trial will continue only until the interim decision stage, if at the initial examination stage, it is known that the dispute has been resolved through a collective agreement. This is far more effective, efficient and fair, and provides legal certainty for the parties.

## 4 Conclusion

The author offers 2 (two) procedural law concepts regarding the implementation of collective agreements. First, a lawsuit for disputes that have been resolved through a cooperative agreement may not be accepted by the court. The judge does not need to process the case further. Still, a file research process is sufficient to explore the party's main problems if the problem is related to one of the parties breaking the promise by referring to the recorded evidence issued by the same court. The Panel of Judges is sufficient to hold a deliberation and only determine with a single judge that the case has been resolved and choose the order for execution. The second concept is that the lawsuit is still being processed. Still, it is only continued until the interim decision stage, if at the initial examination stage, it is known that the dispute has been resolved through a collective agreement. This is far more effective, efficient and fair, and provides legal certainty for the parties.

## Declarations

### Author contribution statement

Salahuddin Gaffara, Agus Mulya Karsonab: Conceived and designed the experiments; Analyzed and interpreted the data; Wrote the paper.

Yani Pujiwatic: Analyzed and interpreted the data; Wrote the paper.

Indra Perwira: Contributed reagents, materials, analysis tools or data; Wrote the paper.

### Funding statement

This research did not receive any specific grant from funding agencies in the public, commercial, or not-for-profit sectors.

### Data availability statement

Data included in article.

### Declaration of interests statement

The authors declare no conflict of interest.

### Additional information

No additional information is available for this paper.
